# Supplementary figures and images for: The effects of exercise on oxidative stress MDA and SOD in patients with type 2 diabetes: a systematic review and meta-analysis
Source: PeerJ. 2025 Aug 21;13:e19814. doi: 10.7717/peerj.19814 (PMC12375296; doi:10.7717/peerj.19814)

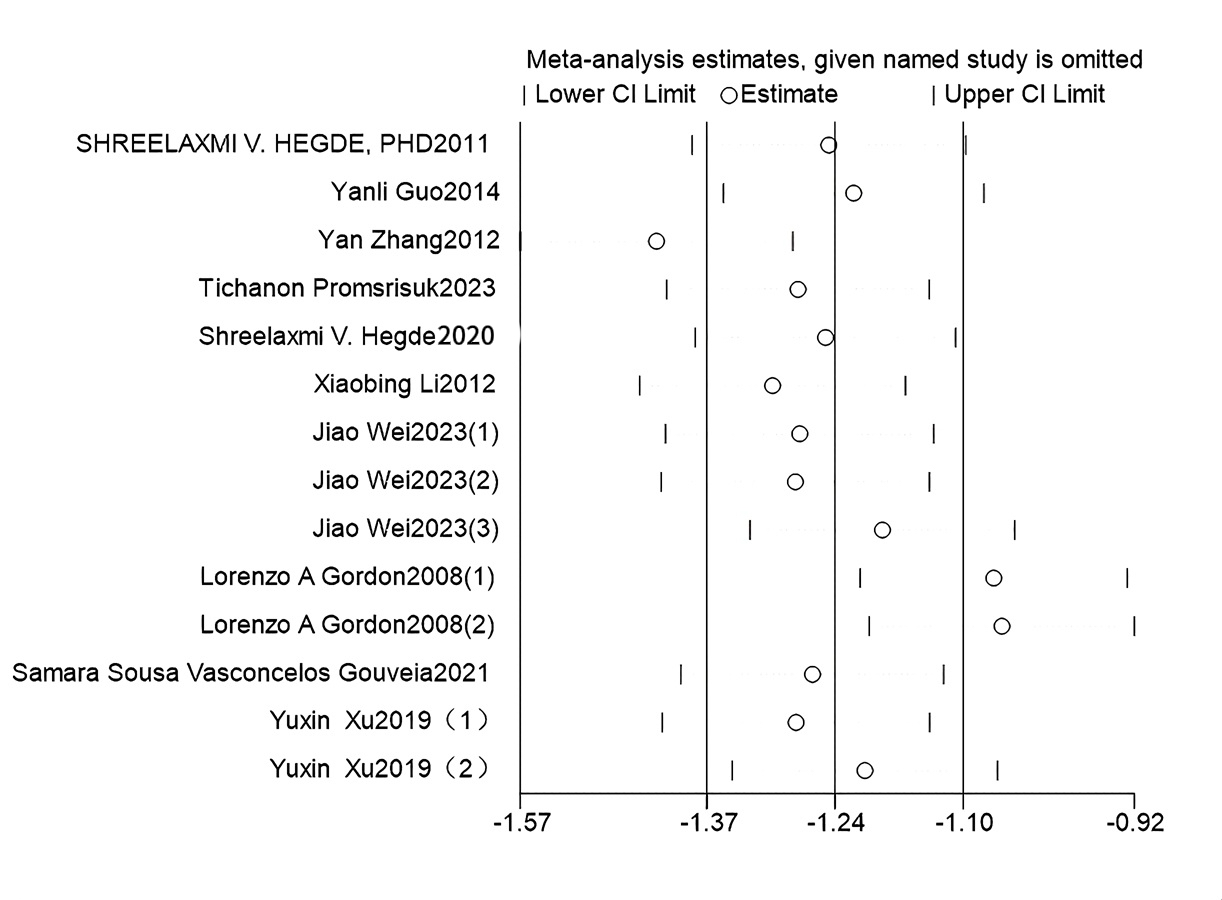

Supplement: Supplemental Information 2 [file peerj-13-19814-s002.jpg]

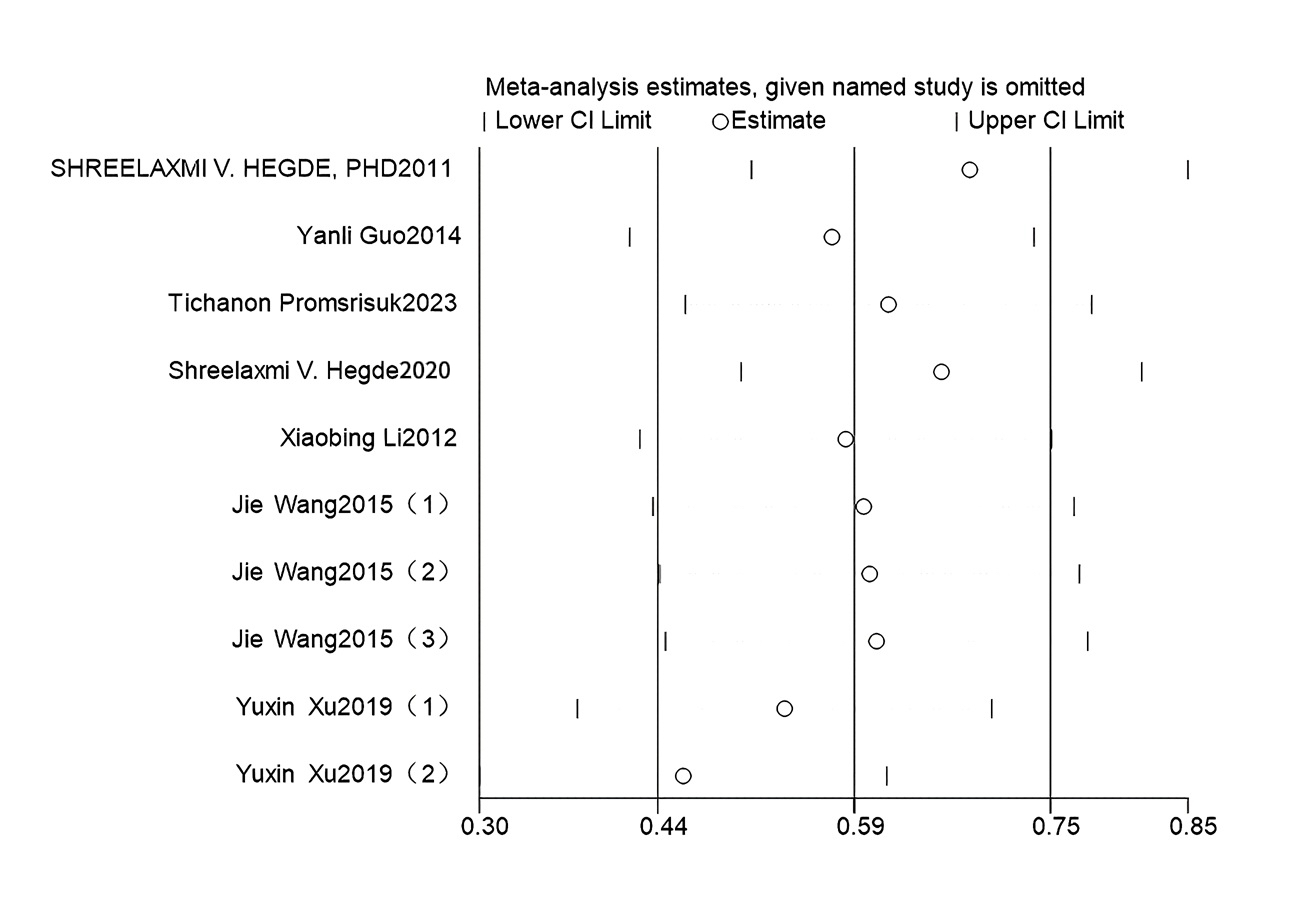

Supplement: Supplemental Information 3 [file peerj-13-19814-s003.jpg]

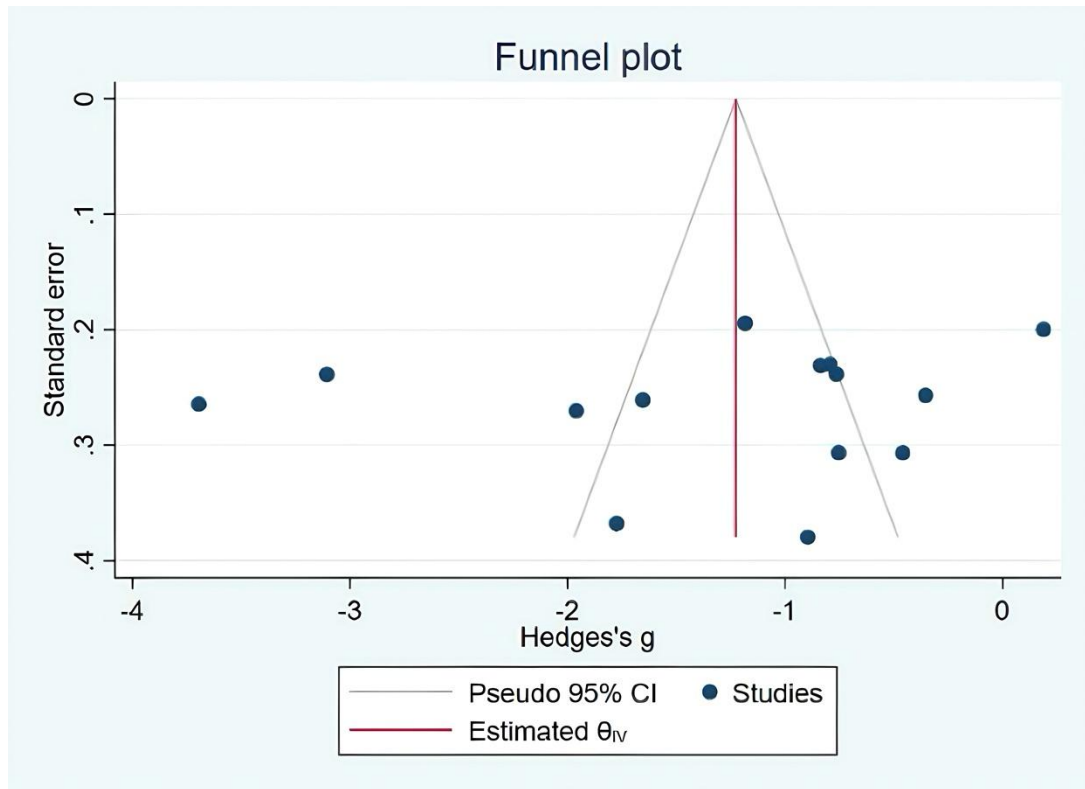

Supplement: Supplemental Information 4 [file peerj-13-19814-s004.pdf]

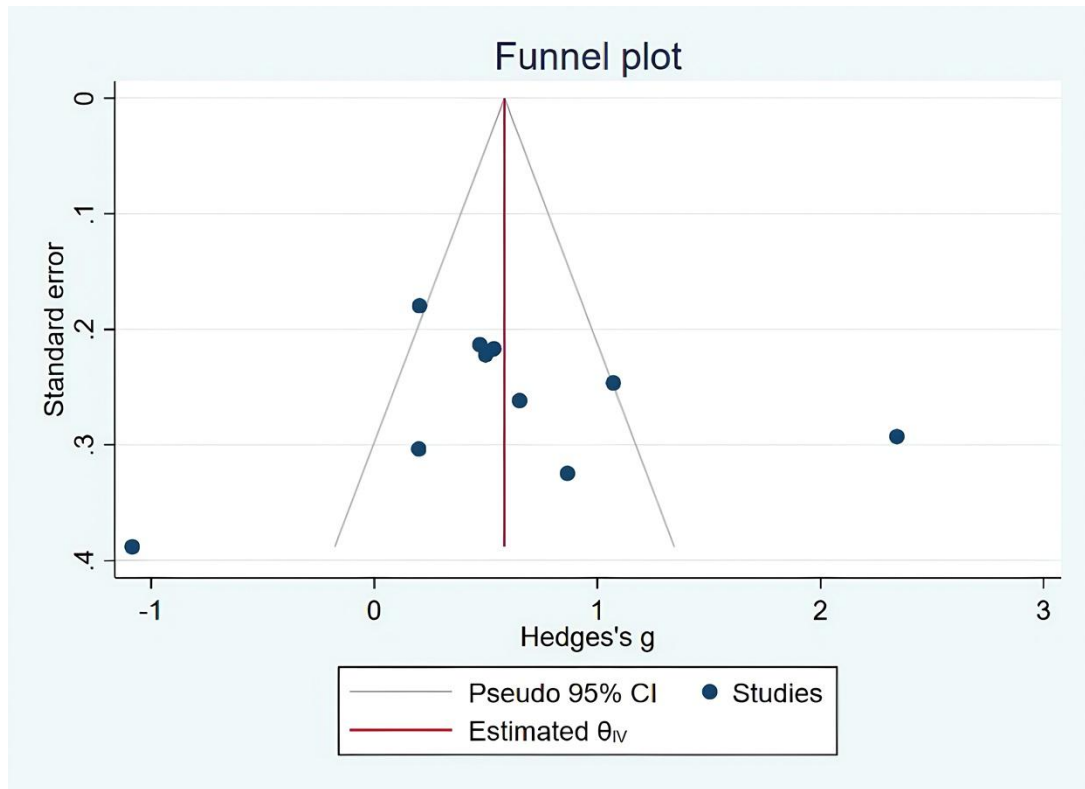

Supplement: Supplemental Information 5 [file peerj-13-19814-s005.pdf]
