# Supplementary material for: The effects of exercise on oxidative stress MDA and SOD in patients with type 2 diabetes: a systematic review and meta-analysis
Source: PeerJ. 2025 Aug 21;13:e19814. doi: 10.7717/peerj.19814 (PMC12375296; doi:10.7717/peerj.19814)
Supplement: Supplemental Information 7 [file peerj-13-19814-s007.docx]

Supplementary document 1 Table 1 PICOS framework for oxidative stress indicators MDA and SOD in patients with Type 2 Diabetes undergoing exercise intervention

| Population | Intervention | Comparison | Outcome | Study design |
| --- | --- | --- | --- | --- |
| Type Diabetes | Exercise  Aerobic exercise  Resistance Exercise  Body and Mind Exercise  Combined Exercise | 1.With or without exercise intervention | Malondialdehyde | Randomized Controlled Trial |
|  |  |  | Super Oxide Dismutase |  |
| Age ≥ 45 years | Intervention prescription  Duration of intervention  Frequency of intervention  Intervention cycle  Place of intervention | 2.Different exercise prescriptions |  |  |
