# Supplementary material for: The effects of exercise on oxidative stress MDA and SOD in patients with type 2 diabetes: a systematic review and meta-analysis
Source: PeerJ. 2025 Aug 21;13:e19814. doi: 10.7717/peerj.19814 (PMC12375296; doi:10.7717/peerj.19814)
Supplement: Supplemental Information 8 [file peerj-13-19814-s008.docx]

Supplementary document1 Table 2 Literature search strategy

| Database | Search steps |
| --- | --- |
| PubMed and The Cochrane Library search strategies | #1“Oxidative Stress” [Mesh] OR “Oxidative” [Title/Abstract] OR “Nitrosative” [Title/Abstract] OR “Nitro Oxidative Stress” [Title/Abstract] OR “Oxidative Injury” [Title/Abstract] OR “Cleavage” [Title/Abstract] OR “Antioxidative” [Title/Abstract] OR “oxidant” [Title/Abstract]  #2 “Sport” [Mesh] OR “Athletic” [Title/Abstract] OR “training” [Title/Abstract] OR “walking” [Title/Abstract] OR “exercise” [Title/Abstract] OR “Tai Chi” [Title/Abstract] OR “yoga” [Title/Abstract] OR “swimming” [Title/Abstract] OR “Anaerobic exercise” [Title/Abstract] OR “Strength Training” [Title/Abstract] OR “Weight Lifting Strengthening Program” [Title/Abstract] OR “Resistance Training” [Title/Abstract] OR “physical activity” [Title/Abstract] OR “Pilates” [Title/Abstract] OR “I Ching” [Title/Abstract] OR “dance” [Title/Abstract]  #3 “Type 2 Diabetes Mellitus” [Mesh] OR “Noninsulin-Dependent Diabetes Mellitus” [Title/Abstract] OR “diabetes type II” [Title/Abstract] OR “T2DM” [Title/Abstract] “diabetes” [Title/Abstract] OR “diabetes mellitus”[Title/Abstract]  #4 Randomized controlled trial [Publication Type] OR “Randomized” [Title/Abstract] OR “controlled” [Title/Abstract] OR “Trial” [Title/Abstract]  #5 #1 AND #2 AND #3 AND #4 |
| Web of Science search strategies | #1 TS=（"Oxidative” OR “Nitrosative” OR “Nitro Oxidative Stress” OR “Oxidative Injury” OR “Cleavage” OR “Antioxidative” OR “oxidant”）  #2 TS=（“Sport” OR “Athletic” OR “training” OR “walking” OR “exercise” OR “baduanjin” OR “Tai Chi” OR “yoga” OR “swimming” OR “aerobics” OR “Anaerobic exercise” OR “Strength Training” OR “Weight Lifting Strengthening Program” OR “Resistance Training” OR “physical activity” OR “Pilates” OR “I Ching” OR “run” OR “dance” OR “bicycle”）  #3 TS=（“Noninsulin-Dependent Diabetes Mellitus” OR “Type 2 Diabetes Mellitus” OR “Type 2 Diabetes” OR “adult onset diabetes” OR “adult onset diabetes mellitus” OR “diabetes mellitus type 2” OR “diabetes mellitus type ii” OR “noninsulin dependent diabetes” OR “noninsulin dependent diabetes mellitus” OR “T2DM” OR “TIIDM” OR “diabetes type 2” OR “diabetes type II” OR “diabetes” OR “diabetic” OR “diabetes”）  #4 TS=（“Randomized controlled trial” OR “Randomized” OR “Controlled” OR “Trial”）  #5 #1 AND #2 AND #3 AND #4 |
| Embase search strategies | #1 “Oxidative Stress” [exp] OR “Oxidative” [ab,ti] OR “Nitrosative”[ab,ti] OR “Nitro Oxidative Stress” [ab,ti] OR “Oxidative Injury” [ab,ti] OR “Cleavage” [ab,ti] OR “Antioxidative” [ab,ti] OR “oxidant” [ab,ti]  #2 “Sport” [exp] OR “Athletic” [ab,ti] OR “training” [ab,ti] OR “walking” [ab,ti] OR “exercise” [ab,ti] OR “Tai Chi” [ab,ti] OR “yoga” [ab,ti] OR “swimming” [ab,ti] OR “Anaerobic exercise” [ab,ti] OR “Strength Training” [ab,ti] OR “Weight Lifting Strengthening Program” [ab,ti] OR “Resistance Training” [ab,ti] OR “physical activity” [ab,ti] OR “Pilates” [ab,ti] OR “I Ching” [ab,ti] OR “dance” [ab,ti]  #3 “Type 2 Diabetes Mellitus” [exp] OR “Noninsulin-Dependent Diabetes Mellitus” [ab,ti] OR “diabetes type II” [ab,ti] OR “T2DM” [ab,ti] OR “diabetes” [ab,ti] OR “diabetes mellitus” [ab,ti]  #4 “Randomized controlled trial” [exp] OR “Randomized” [ab,ti] OR “Controlled” [ab,ti] OR “Trial” [ab,ti]  #5 #1 AND #2 AND #3 AND #4 |
| China Knowledge Network Search Strategy | Topic = (People with Diabetes + Type 2 Diabetes + Non-Insulin Dependent People) AND Topic = (Exercise + Exercise + Mind & Body + Training + Aerobic + Anaerobic + Resistance Exercise + Tai Chi + Ba Duan Jin + Yoga + Pilates + Walking + Swimming + Running + Cycling + Balls) AND Topic = (Oxidative Stress + Oxidative Stress Response + Indicators of Oxidative Stress + State of Oxidative Stress) |
| Wanfang, Wipu search strategy | Topic = (Diabetics OR Type 2 Diabetes OR Non-Insulin Dependent) AND Topic = (Exercise OR Exercise OR Mind-Body Exercise OR Training OR Aerobic Exercise OR Anaerobic Exercise OR Resistance Exercise OR Tai Chi OR Baduanjin OR Yoga OR Pilates OR Walking OR Swimming OR Running OR Cycling) AND Topic = (Oxidative Stress OR Oxidative Stress Response OR Oxidative Stress OR Oxidative Stress Indicator OR Oxidative Stress Status) |
