# Supplementary material for: The effects of exercise on oxidative stress MDA and SOD in patients with type 2 diabetes: a systematic review and meta-analysis
Source: PeerJ. 2025 Aug 21;13:e19814. doi: 10.7717/peerj.19814 (PMC12375296; doi:10.7717/peerj.19814)
Supplement: Supplemental Information 9 [file peerj-13-19814-s009.docx]

Supplementary document1 Table 3 Basic information on the included literature

| Inclusion of literature | Country/area | Sample size/pc | Age/years | Intervention | Cyclicality | Frequency | Time/day | Assessment tools |
| --- | --- | --- | --- | --- | --- | --- | --- | --- |
|  |  | （T/C） | （T/C） | （T/C） |  |  |  |  |
| SHREELAXMI V. HEGDE,PHD2011 | India | 60/63 | 59.8±9.9/57.5±8.9 | Yoga/Regular | 12 weeks | ≥3days/week | —— | 1. ② |
| Guo yanli2014 | China | 20/20 | 51.2±4.7/53.1±5.2 | Aerobic/Regular | 10 weeks | 3-5days/week | 30-45min |  |
| Zhang yan2012 | China | 50/49 | 51.2/50.8 | Walking/Routine | 8 weeks | ≥5days/week | 30min |  |
| Tichanon Promsrisuk2023 | Thailand | 21/21 | 64.29±3.74/64.86±3.55 | Resistance + Yoga/Routine | 12 weeks | 5days/week | 40min | 1. ② |
| ShreelaxmiV.Hegde2020 | India | 14/15 | 46.50±13.03/44.67±9.57 | Yoga/Regular | 12 weeks | 5days/week | 75-90min | 1. ② |
| Li xiaobing2012 | China | 30/30 | 57.3±10.3/57.3±10.3 | Tai Chi/Regular | 8 weeks | 7days/week | 45min | 1. ② |
| Wang jie2015（1） | China | 50/50 | 63.91±4.12/64.51±3.07 | Ba Duan Jin/Regular | 24 weeks | 3days/week | 60min |  |
| Wang jie 2015（2） | China | 50/50 | 63.79±5.01/64.51±3.07 | Relaxation work/ Regular | 24 weeks | 5days/week | 60min |  |
| Wang jie 2015（3） | China | 50/50 | 64.63±3.73/64.51±3.07 | Ba Duan Jin + Relaxation work/Regular | 24 weeks | 5days/week | 60min |  |
| Wei jiao2023（1) | China | 40/40 | 52.35±3.42/52.14±3.28 | Aerobic/Regular | 4 weeks | 7days/week | 30-60min | 1. ② |
| Wei jiao 2023（2) | China | 40/40 | 51.53±3.25/52.14±3.28 | Resistance/Regular | 4 weeks | 7days/week | —— | 1. ② |
| Wei jiao 2023（3) | China | 40/40 | 52.06±3.37/52.14±3.28 | Aerobic + Resistance/Regular | 4 weeks | 7days/week | —— | 1. ② |
| LorenzoAGordon2008(1) | Jamaican | 77/77 | 64/63.6 | Yoga/Regular | 24 weeks | 1days/week | 120min |  |
| LorenzoAGordon2008(2) | Jamaican | 77/77 | 63.9/63.6 | Aerobic/Regular | 24 weeks | 1days/week | 120min |  |
| Samara Sousa VasconcelosGouveia2021 | Brazilian | 22/22 | 59.1±7.4/63.4±9 | Yoga/Stretch and Relax | 8 weeks | 7days/week | 60min |  |
| Xu yuxin2019（1） | China | 38/36 | 65.41±5.01/66.32±5.35 | Aerobic/General Exercise Instruction | 24 weeks | 5days/week | 30min | 1. ② |
| Xu yuxin2019（2） | China | 42/36 | 65.46±4.98/66.32±5.35 | Aerobic + Resistance / General Exercise Instruction | 24 weeks | 5days/week | —— | 1. ② |

Note: T: intervention group C: control group $①$ Malondialdehyde (MDA)

1. Superoxide dismutase (SOD)
