# Supplementary material for: The effects of exercise on oxidative stress MDA and SOD in patients with type 2 diabetes: a systematic review and meta-analysis
Source: PeerJ. 2025 Aug 21;13:e19814. doi: 10.7717/peerj.19814 (PMC12375296; doi:10.7717/peerj.19814)
Supplement: Supplemental Information 10 [file peerj-13-19814-s010.docx]

Supplementary document1 Table 4 Methodological Quality Assessment

| Inclusion of studies | Eligibility criteria | Random allocation | Assignment hiding | Baseline similarity | Blindness of the study population | Therapist blindness | | Outcome assessment of blindness | | Participation rate > 85% | | Intention-to-treat analysis | | Intergroup analysis of statistical results | Point measurements and difference values | totals | |
| --- | --- | --- | --- | --- | --- | --- | --- | --- | --- | --- | --- | --- | --- | --- | --- | --- | --- |
| SHREELAXMI VHEGDE, PHD2011 | 1 | 1 | 0 | 1 | 0 | 0 | 0 | | 1 | | 1 | | 1 | | 1 | 6 |  |
| Guo yanli2014 | 1 | 1 | 0 | 1 | 0 | 0 | 0 | | 1 | | 1 | | 1 | | 1 | 6 |  |
| Zhang yan2012 | 1 | 1 | 0 | 1 | 0 | 0 | 0 | | 1 | | 1 | | 1 | | 1 | 6 |  |
| Tichanon Promsrisuk2023 | 1 | 1 | 0 | 1 | 1 | 0 | 1 | | 1 | | 1 | | 1 | | 1 | 8 |  |
| Shreelaxmi V. Hegde  2020 | 1 | 1 | 0 | 1 | 0 | 0 | 0 | | 1 | | 1 | | 1 | | 1 | 6 |  |
| Li Xiaobing2012 | 1 | 1 | 0 | 1 | 0 | 0 | 0 | | 1 | | 1 | | 1 | | 1 | 6 |  |
| Wang jie2015 | 1 | 1 | 0 | 1 | 0 | 1 | 0 | | 1 | | 1 | | 1 | | 1 | 7 |  |
| Wei jiao2023 | 1 | 1 | 0 | 1 | 0 | 0 | 0 | | 1 | | 1 | | 1 | | 1 | 6 |  |
| Lorenzo  A Gordon  2008 | 1 | 1 | 0 | 1 | 0 | 1 | 1 | | 1 | | 1 | | 1 | | 1 | 8 |  |
| Samara Sousa Vasconcelos Gouveia  2021 | 1 | 1 | 0 | 1 | 0 | 0 | 1 | | 1 | | 1 | | 1 | | 1 | 7 |  |
| Xu yuxin2019 | 1 | 1 | 0 | 1 | 0 | 0 | 0 | | 1 | | 1 | | 1 | | 1 | 6 |  |
