# Supplementary material for: The effects of exercise on oxidative stress MDA and SOD in patients with type 2 diabetes: a systematic review and meta-analysis
Source: PeerJ. 2025 Aug 21;13:e19814. doi: 10.7717/peerj.19814 (PMC12375296; doi:10.7717/peerj.19814)
Supplement: Supplemental Information 11 [file peerj-13-19814-s011.docx]

Type 2 Diabetes Mellitus (T2DM) is a chronic metabolic disorder that requires long-term management and control. It is characterized by insulin resistance, lipid accumulation, impaired glycogen synthesis, and mitochondrial dysfunction, which prevent glucose from being transported into the body’s cells for storage in response to insulin stimulation. The pathogenesis of T2DM is caused by the interaction between insulin dysfunction and insulin resistance. Currently, approximately 537 million adults worldwide suffer from diabetes, with T2DM accounting for over 90% of these cases. It is estimated that by 2040, the number of people with diabetes will rise to 642 million. The incidence of diabetes is steadily increasing worldwide, leading to significant health risks and economic burdens for patients. Therefore, preventing and mitigating the onset and progression of T2DM is an urgent issue that needs to be addressed.

Oxidative stress refers to the imbalance between the production of reactive oxygen species (ROS) and reactive nitrogen species (RNS) and the body’s antioxidant defense system, leading to the excessive production of ROS and RNS, which causes damage to biological macromolecules such as cellular tissues, proteins, and nucleic acids. A key indicator of oxidative stress is the increase in malondialdehyde (MDA) concentration and the decrease in superoxide dismutase (SOD) levels. This leads to damage to pancreatic β-cell function and peripheral insulin resistance, triggering the development of diabetes. In severe cases, it can lead to complications such as diabetic neuropathy, diabetic retinopathy, and diabetic cardiovascular disease, making oxidative stress a critical factor in the onset and progression of T2DM.
