# Supplementary material for: The effects of exercise on oxidative stress MDA and SOD in patients with type 2 diabetes: a systematic review and meta-analysis
Source: PeerJ. 2025 Aug 21;13:e19814. doi: 10.7717/peerj.19814 (PMC12375296; doi:10.7717/peerj.19814)
Supplement: Supplemental Information 6 [file peerj-13-19814-s006.pdf]

Exercise for Type 2 Diabetes

Patient or population: Type 2 Diabetes
Settings:
Intervention: Exercise

Outcomes	Illustrative comparative risks* (95% CI)		Relative effect (95% CI)	No of Participants (studies)	Quality of the evidence (GRADE)	Comments
	Assumed risk	Corresponding risk				
	**Control**	**Exercise**				
**MDA**		The mean mda in the intervention groups was **1.29 standard deviations lower** (0 higher to 0.71 lower)		1137 (10 studies)	⊕⊕⊕⊖ **moderate**	
**SOD**		The mean sod in the intervention groups was **0.59 standard deviations higher** (0.17 to 1.01 higher)		705 (7 studies)	⊕⊕⊕⊕ **high**	

*The basis for the **assumed risk** (e.g. the median control group risk across studies) is provided in footnotes. The **corresponding risk** (and its 95% confidence interval) is based on the assumed risk in the comparison group and the **relative effect** of the intervention (and its 95% CI).

CI: Confidence interval;

GRADE Working Group grades of evidence
High quality: Further research is very unlikely to change our confidence in the estimate of effect.
Moderate quality: Further research is likely to have an important impact on our confidence in the estimate of effect and may change the estimate.
Low quality: Further research is very likely to have an important impact on our confidence in the estimate of effect and is likely to change the estimate.
Very low quality: We are very uncertain about the estimate.
